# Natural Product Identification and Molecular Docking Studies of Leishmania Major Pteridine Reductase Inhibitors

**DOI:** 10.3390/ph18010006

**Published:** 2024-12-24

**Authors:** Moses N. Arthur, George Hanson, Emmanuel Broni, Patrick O. Sakyi, Henrietta Mensah-Brown, Whelton A. Miller, Samuel K. Kwofie

**Affiliations:** 1 Department of Parasitology, Noguchi Memorial Institute for Medical Research (NMIMR), College of Health Sciences (CHS), University of Ghana, Legon, Accra P.O. Box LG 581, Ghana; marthur3@ur.rochester.edu (M.N.A.); gehanson@noguchi.ug.edu.gh (G.H.); 2 Department of Biomedical Engineering, University of Rochester, Rochester, NY 14627, USA; 3 Department of Medicine, Loyola University Medical Center, Maywood, IL 60153, USA; ebroni@luc.edu; 4 Department of Chemistry, School of Physical and Mathematical Sciences, College of Basic and Applied Sciences, University of Ghana, Legon, Accra P.O. Box LG 56, Ghana; patrick.sakyi@uenr.edu.gh; 5 Department of Chemical Sciences, School of Sciences, University of Energy and Natural Resources, Sunyani P.O. Box 214, Ghana; 6 West African Centre for Cell Biology of Infectious Pathogens, Department of Biochemistry, Cell and Molecular Biology, College of Basic and Applied Sciences, University of Ghana, Accra P.O. Box LG 54, Ghana; hemensah-brown@ug.edu.gh; 7 Department of Molecular Pharmacology & Neuroscience, Loyola University Medical Center, Maywood, IL 60153, USA; 8 Department of Biomedical Engineering, School of Engineering Sciences, College of Basic & Applied Sciences, University of Ghana, Legon, Accra P.O. Box LG 77, Ghana

**Keywords:** natural products, molecular docking, molecular dynamics simulations, ADMET, MM/PBSA, *Leishmania*, Pteridine reductase inhibitors

## Abstract

**Background/Objectives**: Pteridine reductase 1 (PTR1) has been one of the prime targets for discovering novel antileishmanial therapeutics in the fight against Leishmaniasis. This enzyme catalyzes the NADPH-dependent reduction of pterins to their tetrahydro forms. While chemotherapy remains the primary treatment, its effectiveness is constrained by drug resistance, unfavorable side effects, and substantial associated costs. **Methods**: This study addresses the urgent need for novel, cost-effective drugs by employing in silico techniques to identify potential lead compounds targeting the PTR1 enzyme. A library of 1463 natural compounds from AfroDb and NANPDB, prefiltered based on Lipinski’s rules, was used to screen against the LmPTR1 target. The X-ray structure of LmPTR1 complexed with NADP and dihydrobiopterin (Protein Data Bank ID: 1E92) was identified to contain the critical residues Arg17, Leu18, Ser111, Phe113, Pro224, Gly225, Ser227, Leu229, and Val230 including the triad of residues Asp181-Tyr194-Lys198, which are critical for the catalytic process involving the reduction of dihydrofolate to tetrahydrofolate. **Results**: The docking yielded 155 compounds meeting the stringent criteria of −8.9 kcal/mol instead of the widely used −7.0 kcal/mol. These compounds demonstrated binding affinities comparable to the known inhibitors; methotrexate (−9.5 kcal/mol), jatrorrhizine (−9.0 kcal/mol), pyrimethamine (−7.3 kcal/mol), hardwickiic acid (−8.1 kcal/mol), and columbamine (−8.6 kcal/mol). Protein–ligand interactions and molecular dynamics (MD) simulation revealed favorable hydrophobic and hydrogen bonding with critical residues, such as Lys198, Arg17, Ser111, Tyr194, Asp181, and Gly225. Crucial to the drug development, the compounds were physiochemically and pharmacologically profiled, narrowing the selection to eight compounds, excluding those with potential toxicities. The five selected compounds ZINC000095486253, ZINC000095486221, ZINC000095486249, 8alpha-hydroxy-13-epi-pimar-16-en-6,18-olide, and pachycladin D were predicted to be antiprotozoal (*Leishmania*) with Pa values of 0.642, 0.297, 0.543, 0.431, and 0.350, respectively. **Conclusions**: This study identified five lead compounds that showed substantial binding affinity against LmPTR1 as well as critical residue interactions. A 100 ns MD combined with molecular mechanics Poisson–Boltzmann surface area (MM/PBSA) calculations confirmed the robust binding interactions and provided insights into the dynamics and stability of the protein–ligand complexes.

## 1. Introduction

Leishmaniasis is counted among the many neglected tropical diseases (NTDs) that affect millions of people in over 90 countries worldwide [1]. An estimated 1 million cases are reported, with a global mortality rate of 70,000 each year [2]. The disease is caused by the trypanosomatid protozoan parasite *Leishmania* and is transmitted through the bite of an infected female phlebotomine sandfly [3]. Approximately 20 *Leishmania* species can infect both humans and animals [4]. Leishmaniasis manifests in three major forms: cutaneous leishmaniasis (CL), visceral leishmaniasis (VL), and mucocutaneous leishmaniasis (ML) [5]. Cutaneous leishmaniasis, the most prevalent among the three, is usually characterized by skin lesions, anemia, fever, etc. The severity depends on the infecting parasite species and the immunity of the host individual [6]. The disease has also been found to be endemic to poor and war-torn communities with poor environmental sanitation and living conditions [7,8]. The Mediterranean region, Asia, the Middle East, North and East Africa, and South and Central America are all endemic for leishmaniasis [9]. The highest prevalence is found in the Indian subcontinent, followed by countries such as Bangladesh, Sudan, South Sudan, Ethiopia, and Brazil [10].

Presently, the lack of efficacious and subsidized drugs is a major hindrance to the extirpation of the disease, especially for individuals living in poor communities. Existing drugs are costly, with prices ranging from USD 30 to USD 1500 [11]. Many individuals in rural regions, grappling with infections, lack the financial means for contemporary medications and turn to herbal remedies to ease their symptoms [12]. Some of the current drugs being used for treatment, which are all from synthetic sources, include amphotericin B, AmBisome, pentavalent antimonial, miltefosine, pentamidine, and paromomycin [13,14]. Amphotericin B, which is associated with differential binding to ergosterol from *Leishmania* membranes, is toxic and expensive [15]. The use of pentavalent antimonials has been curtailed because of side effects, toxicity, and the development of resistance, particularly in India, Bangladesh, and Nepal [16,17]. Similarly, pentamidine demonstrated substantial toxicity when tested against VL [18]. Miltefosine has been recently reported to have a high risk of clinical failures, frequent gastrointestinal side effects, and teratogenic consequences [19]. To reduce these effects, a combination of these drugs is being implemented to decrease the treatment duration, reduce the risk of drug resistance, and improve safety by reducing the dosage of toxic drugs [20,21].

There are still questions about the effectiveness, ease of quality control, stringent regulations, quick effects, toxicity, and efficacy of the current drugs [22]. Due to this, natural products have become a preferable source in the search for efficacious drugs against the disease. Compounds derived from plants have a long history of clinical use, and they are more tolerated and accepted by patients [23]. In the last four decades, approved drugs have been directly or indirectly derived from natural products [22,24]. Three chroman-4-one scaffold flavanones (compounds **1**–**3**) synthesized by Di Pisa et al. [25] were found to inhibit LmPTR1 and TbPTR1 in vitro to differing degrees. Of all the compounds, compound **1** was discovered to be the most powerful, with low toxicity. In vitro tests revealed that hydrazine-coupled pyrazole derivatives were 174 and 2.6 times more potent against LmPTR1 than miltefosine and amphotericin B deoxycholate, respectively [26].

*Leishmania* species, the causative protozoan of leishmaniasis, are auxotrophic trypanosomatids that require reduced pterins and folates for cell growth. Pterins are used by this species to biosynthesize neurotransmitters [27,28,29]. Research conducted by Nare et al. to investigate new chemotherapy for Leishmaniasis by reviewing the literature on the advancement of the metabolism of pteridines in *Leishmania* revealed that Pteridine reductase, an enzyme expressed by these auxotrophs, is responsible for the folate reduction and growing resistance to antifolates by the dihydrofolate reductase and thymidylate synthase (DHFR-TS) complex [29]. DHFR-TS is a very ambitious drug target for the treatment of the disease. It synthesizes folates that serve as co-factors in the synthesis of deoxyribonucleic acid (DNA) nucleotides needed for DNA replication and other growth functions by converting deoxyuridine monophosphate to deoxythymine monophosphate. However, classical antifolates such as methotrexate, trimethoprim, and pyrimethamine have all proven futile against DHFR-TS because of the presence of Pteridine reductase in *Leishmania*. Pteridine reductase is overexpressed in the presence of antifolate to mitigate the introduced toxicities, hence serving to reduce folates in response to the inhibited DHFR-TS complex. This bypass mechanism provided by PTR1 is complemented by DHFR-TS, as it may also be overexpressed in the presence of antileishmanial drugs to act as a bypass for PTR1 by reducing pterins in place of the inhibited PTR1. *L. donovani* has been shown to convert folate to biopterin to be reduced by DHFR-TS in vitro, suggesting this as an alternate biopterin reduction pathway to reduce the needed pterins in adverse conditions (i.e., inhibition of PTR1 only). Efficient chemotherapy for treating *Leishmania* would ideally target both enzymes [30].

**Figure 1:**
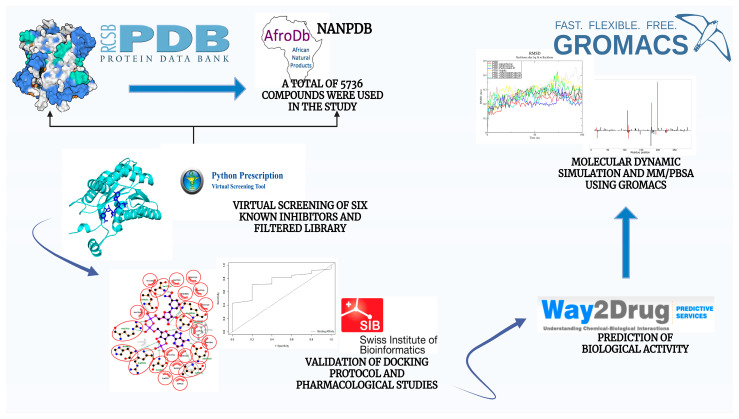
Flow diagram depicting the methods and tools used in this study to predict novel inhibitors against the LmPTR1 enzyme. The first step involves the retrieval of the target protein from the protein databank as well as the compounds from the Zinc database. The protein was then prepared while the ligand library was curated before carrying out the virtual screening using AutoDock Vina. The first step was the selection of the best structure and the compounds for study, followed by 1463 prefiltered substances being screened against the PTR1. Next, the biological activities of the hits were elucidated through binding interactions and pharmacological studies. Finally, molecular dynamics and MM/PBSA of the predicted lead compounds were conducted, involving a run of 100 nanoseconds to assess the stability of the leads and monitor any conformational changes.

Although chemotherapy is the primary treatment for human leishmaniasis, its effectiveness is constrained by drug resistance, unfavorable side effects, and substantial associated costs [31]. Current treatments involve the use of repurposed drugs [9]. This study aimed to discover novel compounds with the potential to disrupt folate and pterin metabolism by targeting the enzyme PTR1 using in silico techniques. The mechanisms of binding between PTR1 and potential inhibitors were characterized through molecular dynamics (MD) simulations combined with molecular mechanics Poisson–Boltzmann surface area (MM/PBSA). Pharmacokinetic and physicochemical profiles, along with biological activity, were analyzed to identify potential antileishmanial leads for next-generation antileishmanial drug design (as depicted in Figure 1).

## 2. Results and Discussion

### 2.1. Analysis of PTR1 Binding Site and Selection of Ligand Library

**Figure 2:**
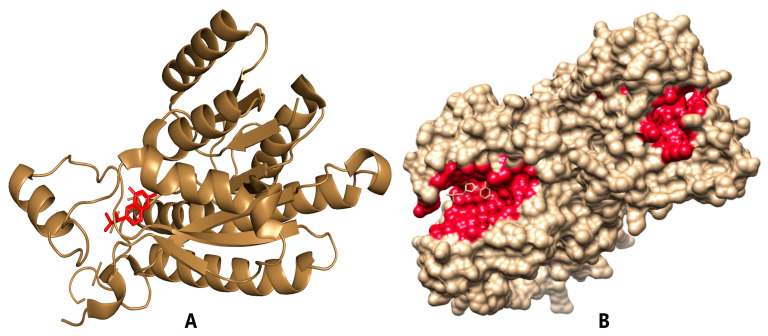
Cartoon representation of chain A of LmPTR1 complexed with the NADP (red) compound in the binding site (**A**). Surface representation of the biological assembly of the LmPTR1 structure comprising all four chains (subunits) complexed with NADP (**B**). The red regions indicate the active sites of the LmPTR1. The images were obtained from PyMOL.

Pteridine reductase 1 (PTR1) is characterized by a 288-residue polypeptide that forms a single α/β domain with short-chain reductase topology [30]. The active site is composed of a solvent-exposed pocket where the cofactor and substrate reside [32]. From the study by Iacono et al. [32], the triad of residues Asp181-Tyr194-Lys198 is crucial for catalyzing the reduction of DHF to tetrahydrofolate by transferring hydride ions and forming hydrogen bond interactions. PTR1 enzymes from several species have been intensively studied as drug targets [25,33,34,35]. This study focused on screening prefiltered natural products from the AfroDb and NANPDB databases against the receptor PTR1 to identify novel antileishmanial and antifolate compounds. LmPTR1 (PDB ID: 1E92) possesses several hydrophobic and hydrophilic binding pockets, where the co-crystallized ligands NADP and DHB bind. According to the crystallographic data, the co-crystallized ligand DHB forms H-bonding and Pi interactions with residues Ser113, Arg17, Arg287, and Ser111, which were included in the analysis. To mimic the key interactions and identify potential lead compounds, the compounds were docked to the active site. The prefiltered library used for the in silico studies contained 1463 compounds. Compounds meeting the selection criteria advanced to the molecular docking stage. Potential novel lead compounds typically exhibit low binding energy (high binding affinity) to the target, acceptable ADME properties, and strong intermolecular interactions between protein receptors and ligands [36]. The controls hardwickiic acid, jatrorrhizine, methotrexate, columbamine, and pyrimethamine (PubChem CIDs 161454, 72323, 126941, 72310, and 4993, respectively), which have demonstrated inhibitory activity, were also incorporated in the molecular docking studies and downstream analysis. The structure of LmPTR1 complexed with NADP is shown in Figure 2.

### 2.2. Validation of Docking Protocol

#### 2.2.1. Alignment and Superimposition

**Figure 3:**
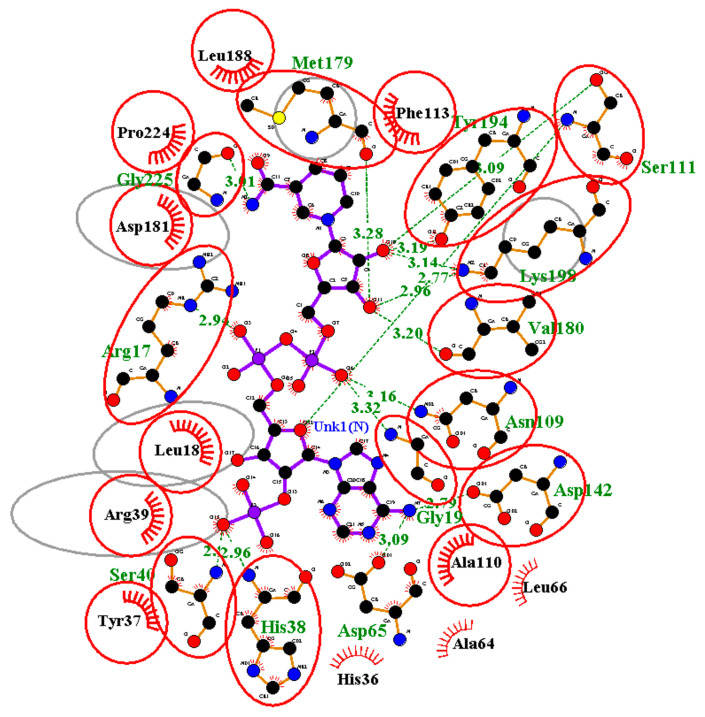
Superimposed LigPlot^+^ image showing the overlapping interactions between the co-crystallized and re-docked ligands (NADP). The overlapped molecular interactions are the residues circled with red. The re-docked NADP reproduced the critical hydrogen bonding and hydrophobic interactions identified in the crystal structure in complex with NADP.

**Figure 4:**
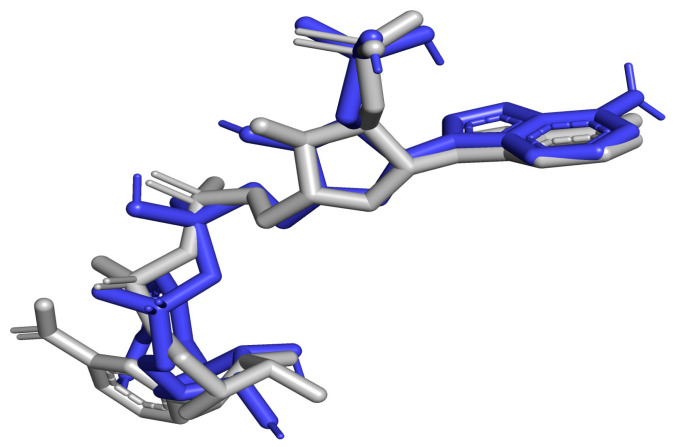
Representation of the superimposition of the re-docked NADP ligand of PTR1 (in blue) with the co-crystallized NADP ligand of PTR1 (in grey) as was shown in PyMOL. RMSD was calculated to be 0.517. This demonstrates that AutoDock Vina is a useful docking tool.

The efficiency and performance of the Autodock Vina are very necessary for the validation of the docking protocol [37,38]. The protocol was validated using the LigAlign [38] script embedded in the PyMOL environment to calculate the RMSD of the superimposed ligands. DHB was removed and re-docked in the same pocket. After the re-docking, it was discovered that both the predicted and experimentally determined binding poses shared mutual interactions with critical residues in the binding pocket. When the re-docked ligand pose of NADP in the structure 1E92 was superimposed on the co-crystallized ligand (NADP), hydrogen bond overlaps involving three critical active site residues (Arg17, Ser111, and Lys198) and other residues (Gly225, Met179, Tyr194, Val180, Asn109, Gly19, His38, Ser40, Asp142, and Asp65) were observed in LigPlot^+^ v.2.2.8 (Figure 3). There were also hydrophobic contact overlaps involving eight residues: Leu18, Tyr37 Ala110, Asp181, Phe113, Leu188, Pro224, and Arg39. The ability of Autodock Vina to reproduce experimentally determined poses is supported and confirmed by these overlaps. The RMSD calculated was 0.517 Å, which is within the 2 Å threshold, regarded as the threshold for good alignment [39]. The superimposition of the re-docked and co-crystallized NADP ligand viewed in Ligplot+ and PyMOL is shown in Figure 3 and Figure 4, respectively.

#### 2.2.2. Receiver Operating Characteristic (ROC) Curve

**Figure 5:**
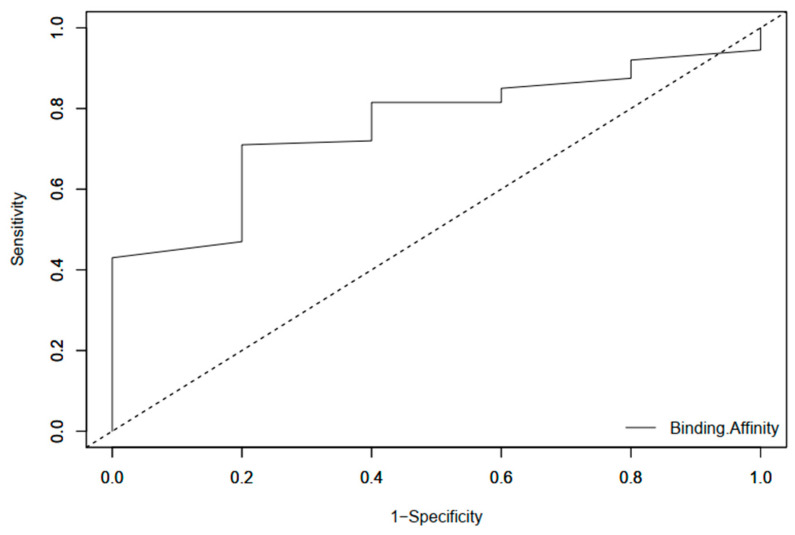
The ROC curve created by screening inhibitors and decoys against the β-OG binding site of the envelope protein. The AUC for this curve is 0.755, which is within acceptable limits.

The ROC curve measures the docking software’s ability to reliably discriminate between active compounds that have been appropriately docked and inactive (decoy) compounds for a particular target [40]. Methotrexate, hardwickiic acid, jatrorrhizine, columbamine, and pyrimethamine were used as actives in validating the docking protocol as they have been referred to in studies as inhibitory ligands [41,42,43,44]. These compounds were subjected to EasyROC simulations to determine the ability of AutoDock Vina to rank known inhibitors highest in a set of decoys generated with DUDe [45] after docking completion. An AUC number near one implies the model can discriminate between actives and decoys, while an AUC value closer to zero indicates that it is less capable of doing so. An AUC of one indicates flawless categorization, whereas 0 indicates no classification capabilities. In the resulting ROC curve (Figure 5), the computed AUC was 0.755, which is satisfactory. This shows that the docking program can distinguish between active and decoy chemicals for the target under consideration. In addition to these validation protocols, several compounds initially identified through molecular docking, particularly with AutoDock Vina, have also demonstrated significant in vitro inhibitory activity against LmPTR1 [26,46,47]. These findings highlight the predictive accuracy of AutoDock Vina and their utility in guiding the experimental validation of potential therapeutic compounds.

### 2.3. Molecular Docking Analysis

**Figure 6:**
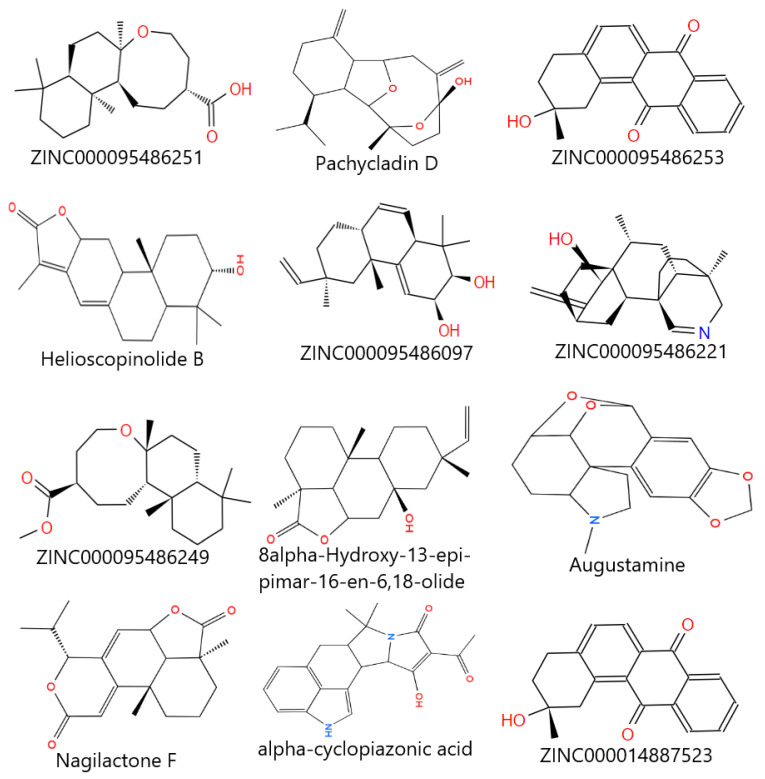
Two-dimensional structures of the top hits after the molecular docking studies.

The PTR1 receptor was observed to be a homotetramer with the active site occurring in each subunit [30]. Al Nasr et al. [48] also adopted this approach when working with PTR1. The cartoon and surface representations of chain A of the protein with NADP in the active site are shown in Figure 2. The same active site was employed for the docking. Molecular docking studies were conducted using 1463 compounds along with five inhibitors (the experimental compounds; methotrexate, jatrorrhizine, pyrimethamine, hardwickiic acid, and columbamine) and the co-crystallized ligand DHB to identify novel potential leads against PTR1. After docking, a stricter cut-off of −8.9 kcal/mol was used instead of −7.0 kcal/mol to differentiate correctly between certain and uncertain protein–ligand interactions [49]. This threshold was used because it was observed to be an average of the binding affinities of the known inhibitors, experimental drugs, and the co-crystallized ligand. Thus, this approach aims to predict more competitive and potentially potent compounds for downstream analysis. A total of 155 compounds were selected since they complied with the −8.9 kcal/mol cut-off. The binding affinities of the compounds and their intermolecular interactions are listed in Table 1. The binding pocket of the PTR1 structure was identified to contain the residues Arg17, Leu18, Ser111, Phe113, Asp181, Tyr194, Lys198, Pro224, Gly225, Ser227, Leu229, and Val230 [30,50,51]; thus, the molecular docking was done in the active site of the protein. Figure 6 shows the two-dimensional structures of the top hits chosen for downstream analysis.

### 2.4. Drug Likeness

The success of molecules intended for drug development hinges largely on their absorption, distribution, metabolism, and elimination (ADME) properties. A drug-like molecule generally exhibits favorable ADME properties [52,53]. Lipinski’s rule of five, Veber’s rule, and the bioavailability score were applied in evaluating these properties. Lipinski’s rule of five is defined by five parameters that can be used to identify orally bioactive compounds with high oral absorption. These parameters are 500 Da or less molecular weight, 5 or less for the octanol–water partition coefficient (lipophilicity), 5 or fewer hydrogen bond donors, 10 or fewer hydrogen bond acceptors, and molar refractivity between 40 Å^2^ and 140 Å^2^ [54]. Fourteen compounds were excluded from downstream analysis due to violation of one parameter of Lipinski’s rule of five. For the known inhibitors, only methotrexate was found to violate the 10 or fewer hydrogen acceptors parameter. All the other inhibitors passed Lipinski’s rule. According to Veber’s rule, compounds with a high chance of being orally bioactive have ten or fewer rotatable bonds and a total polar surface area (TPSA) less than or equal to 140 Å^2^ (optimally between 110 Å^2^ and 140 Å^2^) [55]. One compound (Altersolanol A) violated Veber’s rule and was therefore excluded from downstream analysis. Methotrexate has a TPSA of 210 Å^2^ and thus violated Veber’s rule. The bioavailability score is also used to distinguish between compounds that are poorly and well absorbed in humans [56]. This study only assessed the bioavailability score of the compounds that passed Lipinski’s rule. All the remaining compounds had a bioavailability score of 0.55 except (−)-eremantholide C and (+)-strigol, which had a bioavailability score of 0.56 because these compounds had a TPSA of 82.06 Å^2^, which falls within the 75 Å^2^ and 150 Å^2^ range.

### 2.5. In Silico ADMET Studies

ADME studies are used to determine whether candidate compounds can be developed into safe, functional, and effective therapeutics depending on their pharmacokinetic properties [57,58]. A drug that is predicted to inhibit all classes of cytochrome P450 will impair the metabolism of other drugs, resulting in drug toxicity [59]. The two most important isoforms, CYP3A4 and CYP2D6, were largely considered. BBB permeability, gastrointestinal absorption (GI absorption), water solubility (Log S), and TPSA are descriptors for analyzing the absorption profile of compounds [60,61]. The blood–brain barrier (BBB) protects the brain from toxic substances in the blood and provides nutrients to brain tissues [62]. P-glycoprotein (P-gp) is an efflux transporter that plays a role in drug absorption, distribution, and elimination in the body [63]. Compounds that are P-gp substrates might be eliminated from cells before the therapeutic effect is achieved, reducing their efficacy. Therefore, in this study, compounds found to be P-gp substrates were eliminated from further analysis. The eight compounds that were taken for further analysis were non-inhibitors of CYP3A4 and CYP2D6, P-gp non-substrates, BBB permeant, and had high GI absorption, as shown in Table 2. This approach was adopted from previous studies targeting the same PTR1 protein [50,64]. Only Nagilactone F violated Lipinski’s rule of 5 (Table 2). Toxicity profiles of the remaining compounds were determined using OSIRIS Data Explorer. The eight shortlisted compounds that were predicted to have non-toxic properties are pachycladin D, Nagilactone F, 8alpha-hydroxy-13-epi-pimar-16-en-6,18-olide, ZINC000095486097, ZINC000095486221, ZINC000095486249, ZINC000095486251, and ZINC000095486253. The inhibitor methotrexate was predicted to possess high mutagenic properties, while Pyrimethamine was predicted to have high mutagenic, tumorigenic, and reproductive effects.

### 2.6. LmPTR1-Ligand Interactions

**Figure 7:**
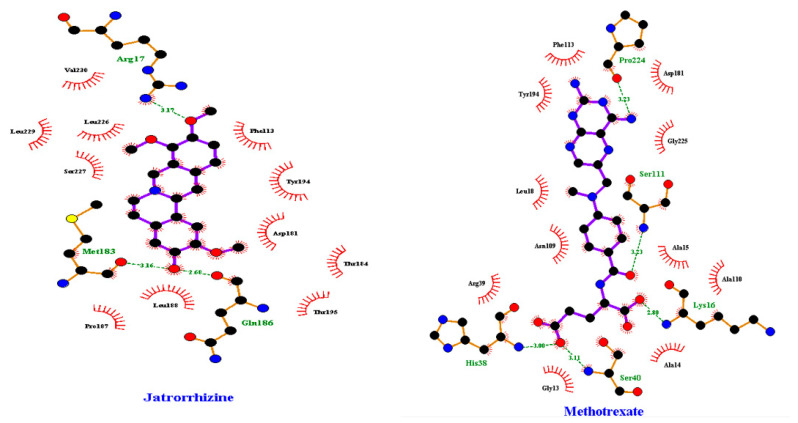
Protein–ligand interaction profiles for LmPTR1 complexed with the inhibitors; methotrexate and jatrorrhizine, depicting both hydrophobic and hydrogen bonds, including their bond length (in green). Carbon is represented by black circles, oxygen by red circles, nitrogen by blue circles, and sulfur by yellow circles. Green-colored residue names interact with hydrogen bonds. Red lines on the ligands relate to hydrophobic links between black residues. The bond lengths observed are all below 3.3 Å.

After ADME/toxicity prediction, LigPlot^+^ was used to elucidate the interactions between the protein and the ligands. For 8alpha-hydroxy-13-epi-pimar-16-en-6,18-olide, three hydrogen bond interactions were observed for the residues Asp181, Tyr194, and Lys198 with bond lengths 3.00, 2.93, and 2.87 Å, respectively, and there were hydrophobic interactions between Arg17, Leu18, Ser111, Phe113, Met183, Leu188, Pro224, Gly225, and Leu226. Additionally, for pachycladin D, residue Arg17 formed a hydrogen bond interaction with a bond length of 3.14 Å, while hydrophobic interactions were formed with Leu18, Ser111, Phe113, Asp181, Leu188, Tyr194, Pro224, Gly225, and Ser227. Nagilactone F formed a hydrogen bond interaction with a bond length of 3.11 with Ser111 Å and hydrophobic interactions with Arg17, Leu18, Asn109, Phe113, Met179, Val180, Asp181, Tyr194, Lys198, Pro224, and Gly225. ZINC000095486251 was also observed to form a hydrogen bond interaction with a bond length of 2.94 with Asn109 Å and hydrophobic interactions with Leu18, Phe113, Val180, Tyr194, Pro224, Gly225, and Ser227. Hydrogen bond interaction with Asn109 with a bond length of 3.16 Å was observed for ZINC000095486221 coupled with hydrophobic interactions between Arg17, Leu18, Ser111, Phe113, Tyr194, Lys198, Met179, Asp181, Pro224, and Gly225. ZINC000095486253 formed hydrophobic interactions with Arg17, Leu18, Phe113, Val180, Asp181, Tyr194, Pro224, Gly225, and Ser227 and hydrogen bond interactions with Gly19 and Asn109 with bond lengths of 2.80 and 3.11 Å (Table 1). ZINC000095486097 and ZINC000095486249 were both observed to form double hydrogen bond interactions with Ser227 and Asn109. ZINC000095486097 formed a hydrogen bond with residues Gly225 and Ser227 with bond lengths of 3.05, 3.06, and 3.18 Å, respectively, and hydrophobic interactions with Arg17, Leu18, Asn109, Tyr194, Phe113, Ser111, Leu226, and Pro224 as shown in Table 1. ZINC000095486249 also formed hydrogen bond interactions with Gly19 and Asn109 with bond lengths of 3.00, 3.15, and 3.18 Å, respectively, and hydrophobic interactions with Gly13, Arg17, Leu18, Ser111, Phe113, Asp181, Pro224, and Gly225. For the known inhibitors, jatrorrhizine was observed to form hydrogen bond interactions of bond lengths of 2.68, 3.17, and 3.16 Å with Gln186, Arg17, and Met183, respectively, and hydrophobic interactions with Phe113, Asp181, Thr184, Pro187, Leu188, Tyr194, Thr195, Leu226, Ser227, Leu229, and Val230 (Figure 7). Additionally, for methotrexate, hydrogen bond interactions with bond lengths of 2.80, 3.00, 3.11, 3.23, and 3.23 Å with Lys16, His38, Ser40, Ser111, and Pro224, respectively, were observed, along with hydrophobic interactions with Gly13, Ala14, Ala15, Leu18, Arg39, Asn109, Ala110, Phe113, Asp181, Tyr194, and Gly225, respectively (Figure 7). All the hydrogen bonds exhibited bond lengths of less than 3.3, with 3.0 being the most common bond length, according to the literature [65].

### 2.7. Structural Similarity Searches of Compounds

The antileishmanial potential of the six potential lead compounds was evaluated through structural searches on PubChem, DrugBank, and http://african-compounds.org/anpdb/ (accessed on 20 November 2023). 8alpha-hydroxy-13-epi-pimar-16-en-6,18-olide, ZINC000095486249, and ZINC000095486251 were found to have structural similarity scores of 0.739, 0.728, and 0.704 with cantharidin (DB12328), respectively. Some studies have concluded that the topical application of cantharidin has the potential to be used in the treatment of *Leishmania* [66,67]. This warrants further in vitro investigations to corroborate the predicted ability of 8alpha-hydroxy-13-epi-pimar-16-en-6,18-olide, ZINC000095486249, and ZINC000095486251 to treat leishmaniasis. Nagilactone F was found to have a structural similarity score of 0.728 with fusidic acid (DB02703). A study has reported using fusidic acid on cutaneous leishmaniasis lesions to prevent the contraction of secondary infections [68]. ZINC000095486253, ZINC000095486097, nagilactone F, 8alpha-hydroxy-13-epi-pimar-16-en-6,18-olide, and pachycladin D were found to be terpenoids or subclasses of terpenoids. ZINC000095486253 had a structural similarity score of 0.774 with bornyl acetate, which belongs to the monoterpenoid molecule subclass under terpenoids. ZINC000095486097 had structural similarity scores of 0.791 and 0.735 with lutein belonging to the tetraterpenoids subclass and ursodiol belonging to the triterpenoids subclass, respectively. Nagilactone F, 8alpha-hydroxy-13-epi-pimar-16-en-6,18-olide, and pachycladin D were identified to be terpenoid molecules from searches conducted on http://african-compounds.org/anpdb/ (accessed on 20 November 2023). Terpenoids are possibly the most diverse group of natural secondary metabolites derived from plants and animals [69]. From the literature, it has been discovered that terpenoids target various cell components and, as a result, have multiple mechanisms of action in the treatment of diseases [69]. Some studies have shown the inhibitory effect of terpenoids on DNA topoisomerase I in *Leishmania* species [70,71] and on the activity of trypanothione reductase in *Leishmania amazonensis* promastigotes [72]. Terpenoids have been predicted to possess antiparasitic properties because of the structural changes induced in *Leishmania* species [73]. Some terpenoids have been observed to induce the effect of increased production of nitric oxide through macrophage activation to kill *L. amazonensis* and L. major species [74,75]. Some monoterpenoids have been reported to cause cell lysis in *L. amazonensis* [76]. Some triterpenoids have been shown in vitro to be effective against *L. infantum* with IC50 values of 8 to 52 µM [77] and IC50 values between 28 and 97 µM [78]. A fingerprint Tanimoto-based similarity search on PubChem revealed that ZINC000095486221 was similar to azitine and isoazitine and that both of the compounds have been shown in vitro to be effective against *L. infantum* [79,80]. Therefore, it is necessary and worthwhile to investigate these potential therapeutic molecules, as they have the potential to be extremely useful in the treatment of *Leishmania*.

### 2.8. Antileishmanial Prediction with PASS

The PASS online server predicts biological activity spectra for 319 types of pharmacological effects, mechanisms of action, and toxic and adverse effects, and it uses a training set of approximately 31,000 biologically active compounds with a mean accuracy of 89% [81]. PASS gives two parameters that specify the significance of the predicted biological activity. The parameters are Pa, which gives information on the probability of activity, and Pi, which gives information on the probability of inactivity. Predicted biological activity with Pa > Pi is deemed pharmacologically noteworthy [82]. This study considered the oxidoreductase inhibitory activity of the compounds since PTR1 belongs to the oxidoreductase enzyme family [83]. Antiprotozoal and antiparasitic activities were considered because it was established that *Leishmania* is a protozoan parasite [84,85]. Additionally, due to the investigated effectiveness of some antifungals [86,87,88], antineoplastic [89,90], antibacterial [91], and anticarcinogenic [92,93] drugs against leishmaniasis, these activities were considered. Compounds ZINC000095486253 (Pa: 0.642 and Pi: 0.012), ZINC000095486221 (Pa: 0.297 and Pi: 0.103), ZINC000095486249 (Pa: 0.543 and Pi: 0.019), 8alpha-hydroxy-13-epi-pimar-16-en-6,18-olide (Pa: 0.431 and Pi 0.038), and pachycladin D (Pa: 0.350 and Pi 0.071) were predicted to be antiprotozoal (*Leishmania*). Only ZINC000095486249 and ZINC000095486253 of the seven shortlisted compounds were predicted to have antileishmanial activity with Pa greater than or equal to 0.5. However, none of the known inhibitors and experimental drugs used in this study was predicted to have antileishmanial activity with Pa greater than or equal to 0.5. Compound ZINC000095486221 was predicted to possess antineoplastic and antifungal properties with Pa: 0.952 and Pi: 0.004 and Pa: 0.190 and Pi: 0.143, respectively. Furthermore, 8alpha-hydroxy-13-epi-pimar-16-en-6,18-olide (Pa: 0.308 and Pi: 0.053) and ZINC000095486249 (Pa: 0.267 and Pi: 0.068) were predicted to be antiparasitic.

### 2.9. Molecular Dynamics (MD) Simulations of Receptor–Ligand Complexes

**Figure 8:**
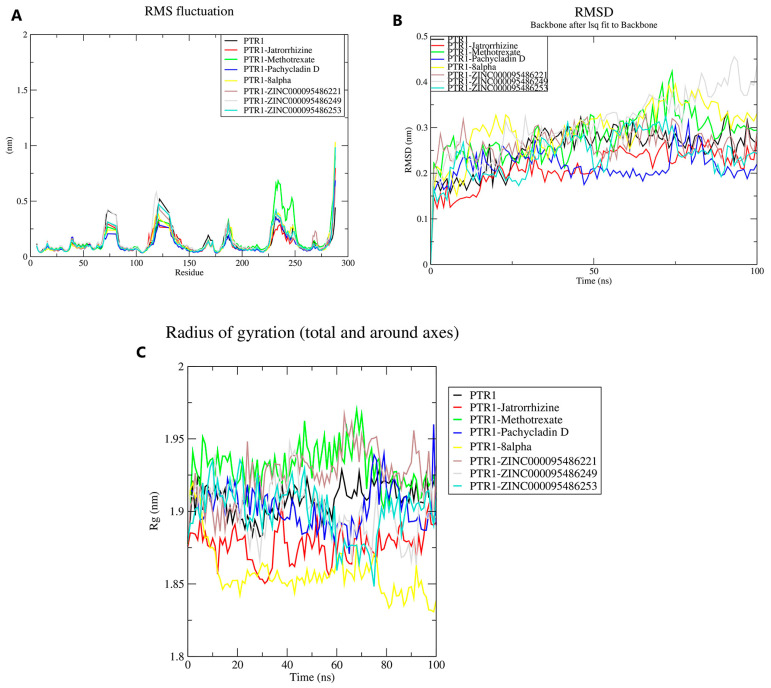
RMSF (**A**), RMSD (**B**), and Rg (**C**) plots of the unbound protein and complexes against time in ns. In all three graphs, black, red, green, blue, yellow, brown, gray, and turquoise represent the unbound protein, jatrorrhizine, methotrexate, pachycladin D, 8-alpha, ZINC000095486221, ZINC000095486249, and ZINC000095486253. 8alpha-hydroxy-13-epi-pimar-16-en-6,18-olide is labeled as 8-alpha in the legend of the plots.

MD simulations were used to assess the stability and conformational nature of the unbound protein and the complex structures with five potential lead compounds and the two known inhibitors methotrexate and jatrorrhizine. The root mean square deviation (RMSD) is acceptable for stability since it is an evaluation of the deviations undergone by the backbone atomic coordinates during simulations. The RMSD over time (ns) showed that the potential lead compounds rose from 0 nm and stabilized around 0.24–0.43 nm. The PTR1–jatrorrhizine and PTR1–-Methotrexate complexes stabilized around the range of 0.14–0.23 nm over time (ns). From approximately 50–75 ns, these complexes remained constant. The PTR1–ZINC000095486221 complex spiked at the beginning of the simulation. However, it became fairly stable from approximately 26 ns to the end (Figure 8). Even with the initial spike, it had fairly identical fluctuations to the unbound protein with a relatively close RMSD. Similarly, the PTR1–ZINC000095486253 complex had similar fluctuations to the unbound protein, albeit with bouts of extreme deviations. On the contrary, the PTR1–ZINC000095486249 complex remained stable over time (ns) around 0.13–0.3 nm until it spiked at around 63 ns, the PTR1–8alpha complex was relatively unstable throughout the simulation, with momentary stability from approximately 24 to 67 ns.

The flexibility of the protein residues was evaluated using the root mean square fluctuation (RMSF). The fluctuations of the individual residues were analyzed over the 100 ns simulation. With few deviations, all five complexes, including 8-alpha and ZINC000095486249, exhibited similar fluctuations to the unbound protein. At the residue positions 65–85, 115–138, and 225–250, high fluctuations were observed.

Lastly, the radius of gyration (Rg) was used to assess the compactness of the bound and unbound protein. A stably folded complex would have a consistent radius of gyration. Except for the PTR1–8alpha complex, the other lead complexes deviated from the unbound protein by at most 0.03 nm. The jatrorrhizine complex stabilized around 1.88 nm, and the ZINC000095486253 complex stabilized around 1.91 nm with a period of instability from 60 to 80 ns (Figure 8). This observation reveals that the jatrorrhizine and ZINC000095486253 complexes remained stable during the simulation.

### 2.10. MM/PBSA Calculations of Receptor–Ligand Complexes

#### 2.10.1. Energy Terms

The molecular mechanics Poisson–Boltzmann surface area (MM/PBSA) approach has been extensively used as an acceptable and definitive binding for calculating the binding free energies of protein–ligand complexes for predicting molecular recognition [94]. The binding free energy of the complexes is the collective energy contributions of the van der Waals, electrostatic, polar solvation, and solvent accessible-surface area (SASA) energies [95]. The MM/PBSA calculations showed that ZINC000095486249 had the lowest binding free energy of −82.349 kJ/mol among the five selected hits. ZINC000095486253, 8alpha, pachycladin D, and ZINC000095486221 also had low binding free energies of −69.190, −62.138, −49.811, and −20.591 kJ/mol. Methotrexate had the highest binding free energy of −10.511 kJ/mol (Table 3). These compounds demonstrated binding affinities superior to the known drug (methotrexate), making them worthy of experimental validation.

#### 2.10.2. Per Residue Energy Decomposition

**Figure 9:**
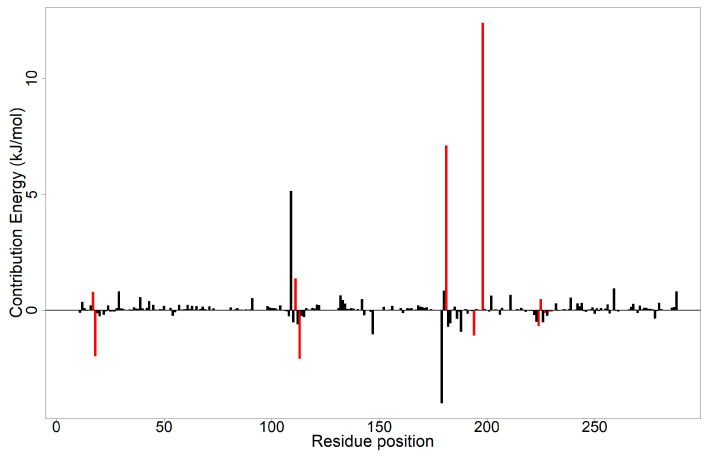
The per residue energy decomposition of the ZINC000095486221 complex. The active site residues are colored red.

Using the MM/PBSA approach, the binding free energies via per residue decomposition can be computed. This is accomplished by examining the interactions of each residue. This facilitates the discovery of the important interactions between residues that contribute to the binding free energy. In line with the literature, residues with energies of more than 5.0 kJ/mol or less than −5.0 kJ/mol were regarded as potentially important active residues that influence the protein–ligand interaction [96]. The per residue energy decomposition computations were performed for each complex (Figure 9).

From the literature, residues Arg17, Leu18, Ser111, Phe113, Asp181, Tyr194, Lys198, Pro224, Gly225, Ser227, Leu229, and Val230 have been identified to be present in the active site [30,50,51]. From the MM/PBSA per residue decomposition computations for the PTR1–ZINC000095486221 complex, it was observed that only Asp181 and Lys198 passed the ±5 kJ/mol threshold with values of 7.1263 and 12.4249 kJ/mol, respectively (Figure 9). Only Asp181 was observed to pass the ±5 kJ/mol threshold with a value of 11.2129 kJ/mol in the PTR1–-Methotrexate complex (Supplementary Figure S1A). The PTR1–8alpha and PTR1–ZINC000095486253 complexes also had one residue, each passing the ±5 kJ/mol threshold, that is, Met136 and Tyr191 with values of −6.9335 and −5.0203 kJ/mol, respectively (Supplementary Figure S1). In the PTR1–Jatrorrhizine, PTR1–Pachycladin D, and PTR1–ZINC000095486253 complexes, no residue contributed energy above the 5 kJ/mol threshold (Supplementary Figure S1).

It is interesting to note while observing the binding interactions after docking, that Asp181 was seen to interact with all five compounds and two known inhibitors with the lowest binding affinities that were analyzed via molecular dynamics simulation. Incidentally, Asp181 was seen to contribute more than 5 kJ/mol to two complexes in the per residue energy computations. This indicates that Asp181 is an essential residue for ligand interaction, requiring experimental verification to ascertain its function.

### 2.11. Future Outlook and Implications of This Study

This study identified molecules with the potential to inhibit the activity of LmPTR1, which could serve as building blocks for the design of novel chemotherapeutics. Furthermore, this study proposed suitable molecules with optimal ADME properties. Because this study is completely computational, additional experimental work will be needed to verify the findings. The binding mechanisms of the predicted lead compounds can be elucidated and adjusted for better binding. Additionally, an assessment of their physiological activity against the *Leishmania* parasite can be conducted. However, challenges such as securing funding for experimental validation exist, as substantial financial resources are required. Moreover, accessing specialized laboratories for testing may hinder progress. Despite these challenges, the predicted molecules may spur efforts worldwide to find potent antileishmanial medications. It is also necessary to investigate the inhibitory potential of the identified biomolecules against PTR1 homologs in the pterin pathway of other *Leishmania* species and trypanosomiasis to identify broad-spectrum pharmacological agents against leishmaniasis and trypanosomiasis. Based on the binding energies and pharmacological profiles demonstrated, combining these predicted compounds with other known drugs can provide a means of combating *Leishmania*. Combination therapy between liposomal amphotericin B and miltefosine, paromomycin, and miltefosine has been developed to shorten treatment duration, increase efficacy, and prevent the emergence of resistance [97].

## 3. Materials and Methods

### 3.1. Protein Preparation and Active Site Characterization

The three-dimensional structure of the LmPTR1 protein was curated from the Protein Data Bank with PDB ID: 1E92 [28,30,85]. To prepare the protein structure for molecular docking, PyMOL (version 2.3.0) was used to remove the compounds nicotinamide-adenine-dinucleotide Phosphate (NADP) and 7,8-dihydrobiopterin (DHB) as well as the water molecules complexed with the structure. Chain A of the protein was used in this study [48,98]. Prior to the molecular docking, energy minimization of the protein was performed using GROMACS (version 5.1.5) with 5000 steps [99,100], and the minimized structure was saved in the format (.gro). The resulting structure was processed in PyMOL and ChimeraX (version 1.16) to prepare the protein for virtual screening [101,102]. The structure saved in .pdb format was converted into AutoDock Vina format (.pdbqt) in PyRx (version 0.8) using the “make macromolecule” feature [103]. Information on the active site of the protein was based on the previous studies focused on LmPTR1 [30,51,104].

### 3.2. Preparation of Ligand Library

A combined prefiltered library of 1463 natural compounds from the African Natural Product Database (AfroDb) and North African Natural Product Database (NANPDB) was used in this study. A total of 812 AfroDb compounds were retrieved from the ZINC database and 4924 NANPDB compounds from the ANPDB database making a total of 5736 natural compounds. Using OSIRIS DataWarrior v5.5.0 [105], the compounds were evaluated based on their conformity to Lipinski’s rule of 5. In addition, the five known and experimental compounds for LmPTR1 inhibition that were used as controls were hardwickiic acid, jatrorrhizine, methotrexate, columbamine, and pyrimethamine with the PubChem CIDs 161454, 72323, 126941, 72310, and 4993, respectively.

### 3.3. Virtual Screening

AutoDock Vina in PyRx was used to screen the filtered library and six known inhibitors against the LmPTR1 protein. The library was imported into Open Babel in PyRx and was minimized by applying the Universal Force Field (Uff) for 200 steps [103,106]. The library was optimized using the conjugate gradient, and the ligands were then converted to “.pdbqt” format. A grid box with dimensions (25, 59.37, 66.13) Å and center (43.37, 39.05, 44.40) Å and the default exhaustiveness of eight were used for the screening.

### 3.4. Docking Validation

The co-crystallized ligand NADP was removed from the complex and re-docked. The re-docked and co-crystallized complexes were superimposed using LigAlign to estimate the root mean square deviation [38]. As an add-on, LigPlot^+^ was used to observe the interaction residues from the complexes for further validation of the docking protocol [107]. In parallel, the Area under the Curve (AUC) function in easyROC [45] was used to calculate the AUC. By using the five (5) known inhibitors as templates, inactives were generated from the DUD-E database [108] and were together docked into the target protein. The AUC value falls within the range of 0 to 1. A 0.5 AUC value indicates no discrimination (i.e., ability to distinguish between positive and negative classes), 1 signifies perfect discrimination, and 0 signifies total misclassification.

### 3.5. Pharmacological Studies/Profiling

The pharmacokinetic and physicochemical properties of each of the selected compounds were analyzed with SwissADME [109]. The physicochemical properties can be used to predict the drug metabolism and toxicity information of the compounds. Molecules that did not meet Lipinski’s and Verber’s criteria were excluded from the analysis. This was done to ascertain the drug-likeness of the predicted compounds.

### 3.6. Binding Mechanism Characterization

LigPlot+ was employed to elucidate protein–ligand interactions with default settings. The best conformations of the selected hits were saved and visualized in PyMOL [107]. LigPlot+ analyzes the molecular interactions, mainly hydrophobic and hydrogen bonds, and generates two-dimensional schematic representations of the interactions in terms of bond types. The hydrogen bonds are characterized by bond length and measured in Angstrom (Å).

### 3.7. Prediction of Biological Activity

The Bayesian-based Prediction of Activity Spectra for Substances (PASS) was used to predict the biological activities of the selected hits [110]. This was also done using the Simplified Molecular Input Line-Entry System (SMILES) files of the hits and uploading them to the PASS online server [111]. It analyzes the chemical structure using the SMILES and generates valuable insights into the possible biological activities of the ligands, to aid the prioritization of promising compounds for further experimental validation [82].

### 3.8. Molecular Dynamics (MD) Simulations and MM/PBSA Calculations of Receptor–Ligand Complexes

GROMACS 2018 [112] was employed to perform MD simulations of the protein–ligand complexes. The CHARMM36 all-atom force field was used to generate the topology of the protein. Generation of ligand topologies was achieved using the CHARMM force field on the CHARMM General Force Field (CGenFF) server (available at https://cgenff.com, accessed on 8 January 2024). For each case under study, complexes were formed from the protein and ligand topologies. In a 1.0 nm cubic box, each complex underwent solvation with a 3-point (TIP3P) water model and neutralization with Na and Cl ions. Using the steepest descent algorithm, each complex was minimized for 50,000 steps. Using the NPT (Number of particles, Pressure, and Temperature) ensemble, the ligands were further restricted after the NVT (Number of particles, Volume, and Temperature). The MD simulation was run for 100 ns under the PME (Particle-Mesh Ewald) after each complex was equilibrated for 100 ps. The simulations unveil the detailed mechanisms of binding as well as direct observation of the motion of biomolecules at the atomic scale [113]. To explore the stability and conformational flexibility of the unbound protein and protein–ligand complexes under study, the root mean square deviation (RMSD), root mean square fluctuation (RMSF), and radius of gyration (Rg) graphs were developed. Finally, G-MM/PBSA was also used to calculate the binding free energies of each complex throughout the 100 ns simulation period, using frames in 0.1 ns steps. The MM/PBSA was used to calculate the binding free energy contribution per residue, and R programming was used to create the graphs. All production MD and MM/PBSA estimations were performed on a Dell EMC high-performance computing cluster at the WACCBIP, University of Ghana, Accra.

## 4. Conclusions

This study enhances current efforts in the development of novel antileishmanials by leveraging natural products as potential inhibitors against PTR1. A collection of 1463 compounds, including five known drugs, were virtually screened against the minimized PTR1. The compounds were evaluated based on their ability to bind firmly into the active site, which is characterized by high binding affinities, protein–ligand interactions, particularly hydrogen and hydrophobic bonding, and satisfying the ADME profiling criteria. In addition, the overall complex stability of the protein–ligand complex and binding free energy was elucidated. In this study, we identified five lead compounds, namely, ZINC000095486221, ZINC000095486249, pachycladin D, ZINC000095486253, and 8alpha-hydroxy-13-epi-pimar-16-en-6,18-olide, which showed substantial binding affinity against LmPTR1 as well interactions with Phe113, Arg17, Ser111, Asp181, Tyr194, Pro224, and Gly225. The molecular dynamics simulations revealed higher stability for the protein–ligand interactions backed by low binding free energy than the methotrexate drug. These compounds can be further exploited for designing novel inhibitors against LmPTR1.

## Figures and Tables

**Table 1:** Binding affinity and molecular interactions of five known inhibitors against PTR1, NADP, and eight hits, including ligand names, PubChem CIDs, and intermolecular bonds.

Ligand	PubChem CID	Binding Affinity (kcal/mol)	Interacting Residues	
			Hydrogen Bonds (Å)	Hydrophobic Interactions
NADP	N/A	−11.0	Arg17(2.89, 2.98), Leu18(3.15), His38(2.85), Arg39(2.73), Ser40(2.85, 3.02), Leu66(2.79), Ser111(2.92, 3.13), Asp142(2.65), Lys198(2.90, 3.00), Ser227(2.65, 3.05)	Gly13, His36, Tyr37, Ala64, Asp65, Asn109, Ala110, Ser112, Met179, Val180, Asp181, Pro224, Gly225, Leu226
Methotrexate	126941	−9.5	Pro224(3.23), Lys16(2.80), His38(3.00), Ser40(3.11), Ser111(3.23)	Gly13, Ala15, Leu18, Tyr37, Arg39, Ala110, Asn109, Phe113, Pro224, Ser227
Jatrorrhizine	72323	−9.0	Arg17(3.17), Met183(3.16), Gln186(2.68)	Phe113, Asp181, Thr184, Pro187, Leu188, Tyr194, Thr195, Leu226, Ser227, Leu229, Val230
Pyrimethamine	4993	−7.3	Val180(3.01, 3.25), Gly225(2.91)	Phe113, Asp181, Leu188, Tyr194, Lys198, Pro224
Columbamine	72310	−8.6	Arg17(3.03), Asp181(2.90)	Ser111, Phe113, Met183, Leu188, Tyr194, Leu226, Tyr283
Hardwickiic acid	161454	−8.1	Arg17(3.07), Leu18(3.10)	Ser111, Phe113, Asn109, Asp181, Tyr194, Lys198
Pachycladin D	46833117	−9.4	Arg17(3.14)	Leu18, Ser111, Phe113, Asp181, Leu188, Tyr194, Pro224, Gly225, Ser227
8alpha-hydroxy-13-epi-pimar-16-en-6,18-olide	273471517	−9.2	Asp181(3.00), Tyr194(2.93), Lys198(2.87)	Arg17, Leu18, Ser111, Phe113, Met183, Leu188, Pro224, Gly225, Leu226
Nagilactone F	181498	−9.1	Ser111(3.11)	Arg17, Leu18, Asn109, Phe113, Met179, Val180, Asp181, Tyr194, Lys198, Pro224, Gly225
ZINC000095485955	N/A	−9.3	Arg17(2.79), Asn109(2.81), Lys198(2.82)	Leu18, Ser111, Met179, Val180, Asp181, Pro224, Gly225, Val228
ZINC000095486097	N/A	−9.0	Gly225(3.05), Ser227(3.06, 3.18)	Arg17, Leu18, Asn109, Tyr194, Phe113, Ser111, Leu226, Pro224
ZINC000014887523	N/A	−9.5	Tyr194(2.86)	Ser111, Phe113, Asp181, Met183, Leu188, Leu226, Leu229
dehydrocardiopetaline	N/A	−9.2	Arg17(2.95, 3.23), Ser111(3.29), Tyr194(2.87)	Leu18, Phe113, Asn109, Val180, Pro224, Gly225, Lys198, Asp181
ZINC000095486221	N/A	−9.9	Asn109(3.16)	Arg17, Leu18, Ser111, Phe113, Tyr194, Lys198, Met179, Asp181, Pro224, Gly225
helioscopinolide B	10335933	−9.2	Lys198(2.88), Met179(3.24), Val180(3.11)	Asn109, Pro224, Leu18, Asp181, Tyr194, Leu188, Gly225, Leu226, Leu229, Phe113, Ser111
alpha-cyclopiazonic acid	135494311	−9.2	Arg17(2.90), Tyr194(3.04)	Phe113, Ser111, Leu229, Val230, Ser227, Leu226, Leu188, Asp181
ZINC000095486249	N/A	−9.3	Gly19(3.00), Asn109(3.15, 3.18)	Gly13, Arg17, Leu18, Ser111, Phe113, Asp181, Pro224, Gly225
ZINC000095486251	N/A	−9.0	Asn109(2.94)	Leu18, Phe113, Val180, Tyr194, Pro224, Gly225, Ser227
ZINC000095486253	N/A	−9.4	Gly19(2.80), Asn109(3.11)	Arg17, Leu18, Phe113, Val180, Asp181, Tyr194, Pro224, Gly225, Ser227
augustamine	5251634	−9.0	Asn109(3.17), Ser111(3.06)	Lys198, Asp181, Tyr194, Phe113, Arg17, Leu18, Val180

**Table 2:** Prediction of ADME properties of selected ligands. The water solubility (ESOL), class of solubility, BBB permeability, gastrointestinal absorption, cytochrome P_450_ inhibition, and P-glycoprotein substrate were computed.

Ligand	MW (g/mol)	TPSA (Å^2^)	ESOL (LogS)	Class of Solubility	BBB Permeability	GI Absorption	CYP3A4 Inhibition	CYP2D6 Inhibition	P-Glycoprotein Substrate	No. of Lipinski’s Rules Violated
*ZINC000095486221*	313.48	32.59	−3.85	Soluble	Yes	High	No	No	No	0
*Pachycladin D*	318.45	38.69	−3.70	Soluble	Yes	High	No	No	No	0
*ZINC000095486253*	336.51	35.53	−5.18	Moderately soluble	Yes	High	No	No	No	0
*8alpha-hydroxy-13-epi-pimar-16-en-6,18-olide*	318.48	46.53	−4.56	Moderately soluble	Yes	High	No	No	No	0
*Nagilactone F*	316.4	52.60	−3.71	Soluble	Yes	High	No	No	No	1
*ZINC000095486097*	302.45	40.46	−4.34	Moderately soluble	Yes	High	No	No	No	0
*ZINC000095486251*	322.48	46.53	−4.89	Moderately soluble	Yes	High	No	No	No	0
*ZINC000095486249*	336.51	35.53	−5.12	Moderately soluble	Yes	High	No	No	No	0

**Table 3:** Contributing energy terms of the molecular mechanics/Poisson–Boltzmann surface area (MM/PBSA) computations for receptor–ligand complexes. Values are shown as the average standard deviation in kJ/mol. The terms include van der Waals, electrostatic, polar solvation, solvent-accessible surface area (SASA), and binding energies.

**Complex**	**van der Waals Energy (kJ/mol)**	**Electrostatic Energy (kJ/mol)**	**Polar Solvation Energy (kJ/mol)**	**SASA Energy (kJ/mol)**	**Binding Energy (kJ/mol)**
PTR1–Jatrorrhizine	−126.865 ± 5.915	−12.087 ± 1.112	80.132 ± 3.705	−14.918 ± 0.674	−73.945 ± 4.145
PTR1–Methotrexate	−117.704 ± 6.462	−47.059 ± 3.179	170.879 ± 8.916	−16.431 ± 0.877	−10.511 ± 4.455
PTR1–Pachycladin D	−68.810 ± 4.603	−4.009 ± 0.664	32.321 ± 4.480	−9.706 ± 0.619	−49.811 ± 4.383
PTR1–8alpha-hydroxy-13-epi-pimar-16-en-6,18-olide	−95.969 ± 5.508	0.346 ± 0.187	45.585 ± 6.444	−11.491 ± 0.677	−62.138 ± 7.084
PTR1–ZINC000095486221	−91.303 ± 4.866	−19.833 ± 1.160	102.029 ± 6.004	−11.441 ± 0.615	−20.591 ± 5.380
PTR1–ZINC000095486249	−105.919 ± 5.065	−0.268 ± 0.099	38.991 ± 6.292	−14.912 ± 0.712	−82.349 ± 6.229
PTR1–ZINC000095486253	−115.144 ± 3.489	−0.342 ± 0.077	62.030 ± 6.824	−16.024 ± 0.493	−69.190 ± 6.923

## Data Availability

The original contributions presented in the study are included in the article/Supplementary Materials, further inquiries can be directed to the corresponding authors.

## Supplementary Materials

The following supporting information can be downloaded at: https://www.mdpi.com/article/10.3390/ph18010006/s1, Figure S1: Supporting information document contains the plots for Molecular mechanics Poisson–Boltzmann surface area (MM/PBSA) plots of binding free energy contribution per residue of protein–ligand complexes (A) PTR1–Methotrexate; (B) PTR1–8alpha-hydroxy-13-epi-pimar-16-en-6,18-olide; (C) PTR1–ZINC000095486253; (D) PTR1–ZINC000095486249; (E) PTR1–Jatrorrhizine; (F) PTR1–Pachycladin D.
