# Supplementary material for: Natural Product Identification and Molecular Docking Studies of Leishmania Major Pteridine Reductase Inhibitors
Source: Pharmaceuticals (Basel). 2024 Dec 24;18(1):6. doi: 10.3390/ph18010006 (PMC11768234; doi:10.3390/ph18010006)
Supplement: Supplementary file 1 [file pharmaceuticals-18-00006-s001.zip › pharmaceuticals-3255358-supplementary.pdf]

Supplementary Information

Natural Product Identification and Molecular Docking Studies of Leishmania Major Pteridine Reductase Inhibitors

A)

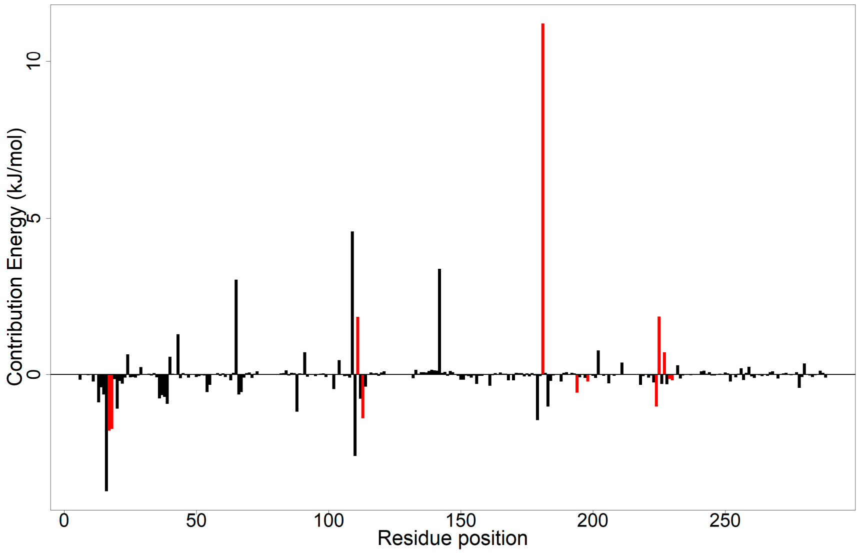

B)

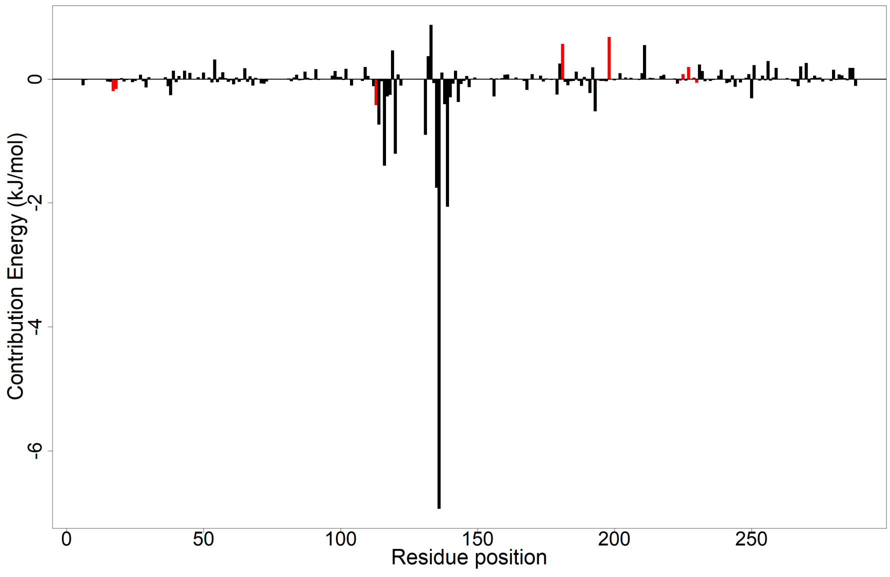

C)

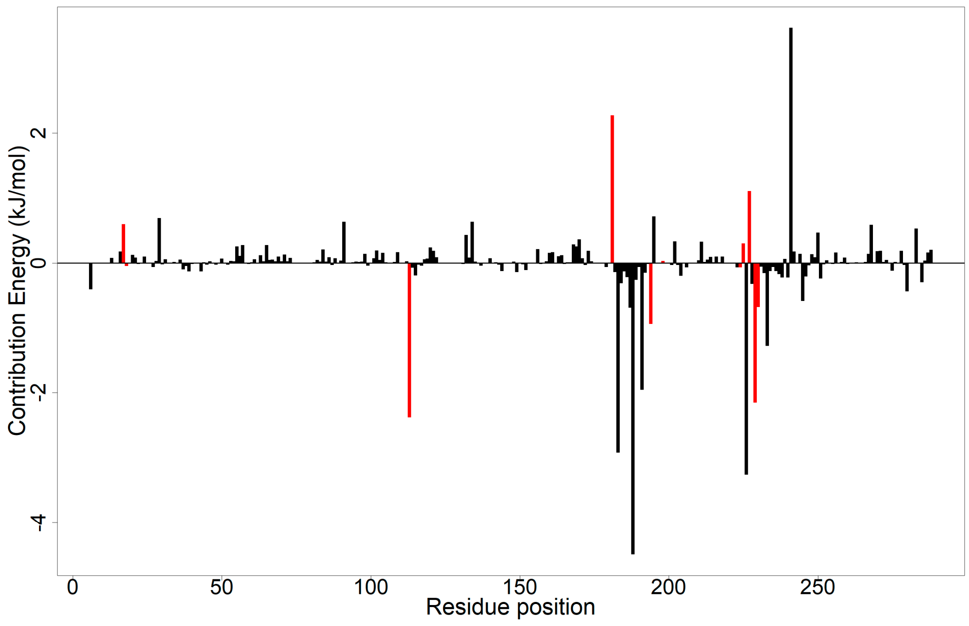
Contribution Energy (kJ/mol)
2
0
-2
-4
0
50
100
150
200
250
Residue position

D)

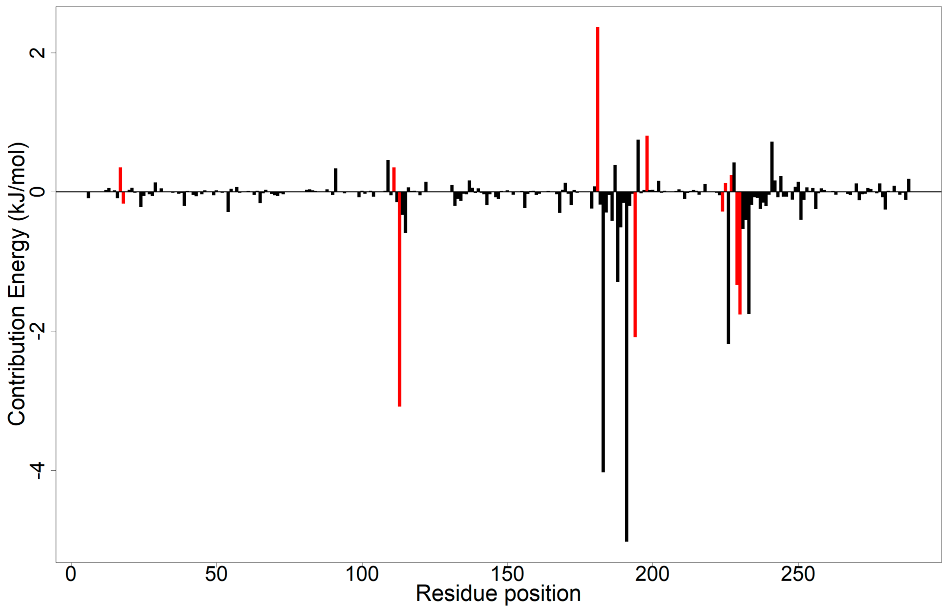
Contribution Energy (kJ/mol)
2
0
-2
-4
0
50
100
150
200
250
Residue position

E)

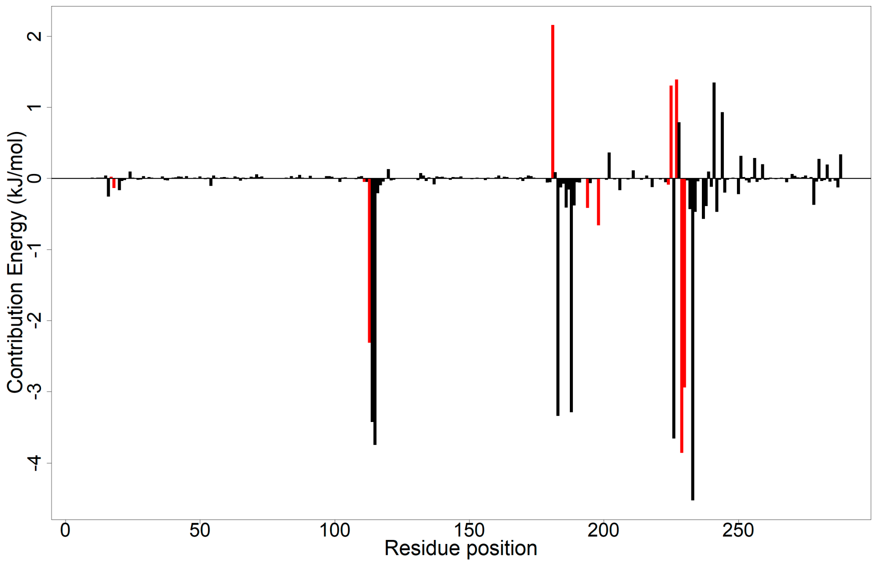

F)

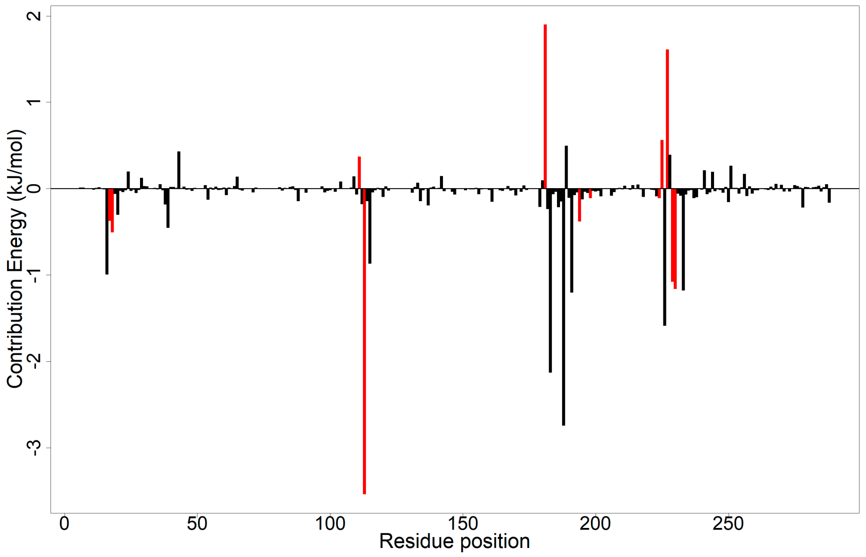

Supplementary Figure S1: Molecular mechanics Poisson-Boltzmann surface area (MM-PBSA) plot of binding free energy contribution per residue of protein-ligand complexes A) PTR1-Methotrexate; B) PTR1-8alpha-hydroxy-13-epi-pimar-16-en-6,18-olide; C) PTR1-ZINC000095486253; D) PTR1-ZINC000095486249; E) PTR1-Jatrorhizine; F) PTR1-Pachycladin D.
